# Renomedullary Interstitial Cell Tumor in the Background of a Renal Oncocytoma: A Case Report

**DOI:** 10.7759/cureus.60531

**Published:** 2024-05-17

**Authors:** Saad Alshathri, Ahmed Alzahrani, Moath Qarmush, Hisham Ghabbani, Faisal Altwijri, Alwaleed Alqarni, Nagoud Ali, Hossam S El-Tholoth

**Affiliations:** 1 College of Medicine, Alfaisal University, Riyadh, SAU; 2 Urology, Prince Sultan Military Medical City, Riyadh, SAU; 3 Urology, King Khalid University Hospital, Riyadh, SAU; 4 Histopathology, Prince Sultan Military Medical City, Riyadh, SAU

**Keywords:** renal cell carcinoma (rcc), renal tumor, oncocytoma, medullary fibroma, renomedullary interstitial cell tumor

## Abstract

Renomedullary interstitial cell tumors (RMICTs) are rare benign renal tumors that arise from the renal medulla. They are rarely symptomatic and are mostly discovered incidentally. Radiologically, their co-presence ipsilaterally in the background of a larger mass introduces a miscellaneous presentation that raises the suspicion of metastatic disease. A characteristic presentation does not exist. Therefore, an individualized, patient-centered approach should be tailored depending on the nature of the presentation. We report the clinical, radiological, and histopathological presentation of a 46-year-old woman presenting with an RMICT in the background of a renal oncocytoma.

## Introduction

Renal oncocytomas are benign epithelial tumors that arise from the intercalated tubular cells of the renal collecting duct. They represent 3% to 7% of all renal tumors and typically demonstrate a consistently benign behavior [1]. Despite the recognition that their growth rate may resemble that of a renal cell carcinoma (RCC), even when they reach large sizes, they generally exhibit a well-defined encapsulation, and they infrequently show invasiveness or association with metastases [1]. Co-presence of a renomedullary interstitial cell tumor (RMICT) previously known as medullary fibromas in the background of a renal oncocytoma is an event that was not yet documented in the literature. RMICTs are rarely symptomatic and generally exhibit a benign presentation [2]. However, differentiating them from other malignant renal masses imposes a great challenge when it comes to clinical practice given their overlap in radiologic, histologic, and immunophenotypic features with RCC resulting in a tendency for overtreatment in the form of an unnecessary partial or radical nephrectomy [2,3]. The enhanced integration of diverse diagnostic methods such as imaging modalities, and histologic and immunophenotypic features contributes to a more precise definition and characterization of renal oncocytomas and RMICTs, however, it necessitates an advanced analysis that is not universally practical or applicable. We report and discuss the clinical, radiological, and histopathological findings of a case presenting with a picture of an oncocytoma with the co-presence of an RMICT in the background.

## Case presentation

**Figure 1:**
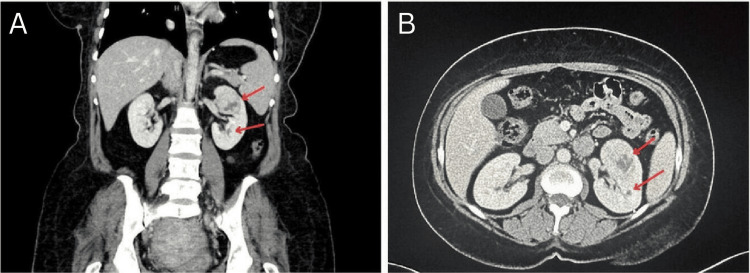
Coronal (A) and axial (B) sections of a contrast abdomino-pelvic CT scan that demonstrates a 4.6 cm left renal arterial enhancing mass with central necrosis with another 0.7 cm hypodense lesion that is posterior to the previously described mass.

A 46-year-old woman was referred to our urology clinic for an incidental 4 cm left upper pole renal lesion on pelvic MRI suggestive of an RCC detected during her gynecological workup. The patient is medically free, a non-smoker, and has no family history of cancer. She is following up with a gynecology duo to refractory uterine fibroids. She was seeking a definitive treatment option, so she elected to undergo a hysterectomy. Pre-operative workup included a pelvic MRI which revealed the incidental renal finding. The patient denied the presence of weight loss, fever, hematuria, or night sweats. Laboratory workup including a complete blood count, renal function tests, urinalysis, and a complete metabolic panel all came back unremarkable. Physical examination revealed no palpable flank masses or tenderness. Staging workup included a contrast chest CT which ruled out pulmonary and mediastinal metastasis as well as a contrast abdominal CT which revealed a 4.6 cm left arterial enhancing mass with central necrosis most likely representing a renal cell carcinoma, another 0.7 cm lesion in the same kidney was also noted which raised suspicion for another focal or metastatic disease (Figure 1).

**Figure 2:**
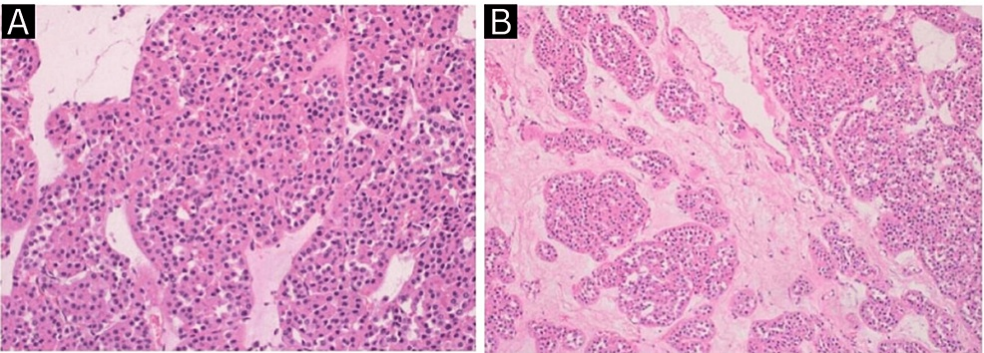
Histopathological findings (A, B) of renal oncocytoma.

**Figure 3:**
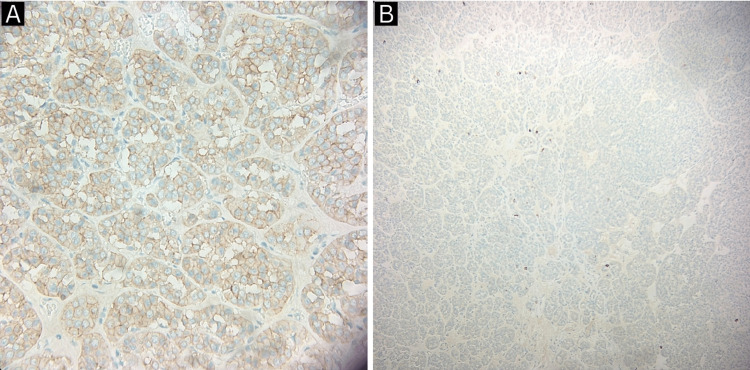
Immunohistochemistry of renal oncocytoma revealing CD 117 +ve cells (A) and cytokeratin 7 +ve cells (B).

Otherwise, no local or distant metastasis was noted in the abdomen or pelvis. Since the metastatic workup came back negative, the patient was counseled and was then booked for a laparoscopic left radical nephrectomy. The operation was unremarkable, the operative time was 120 minutes, and the estimated blood loss was 75 ml. Histopathological and immunohistochemical evaluation of the kidney revealed a well-defined, round, brown, soft tissue mass located in the upper pole measuring 4.0 x 3.5 x 3.0 cm extending to the renal sinus fat consistent with an oncocytoma (Figures 2, 3).

**Figure 4:**
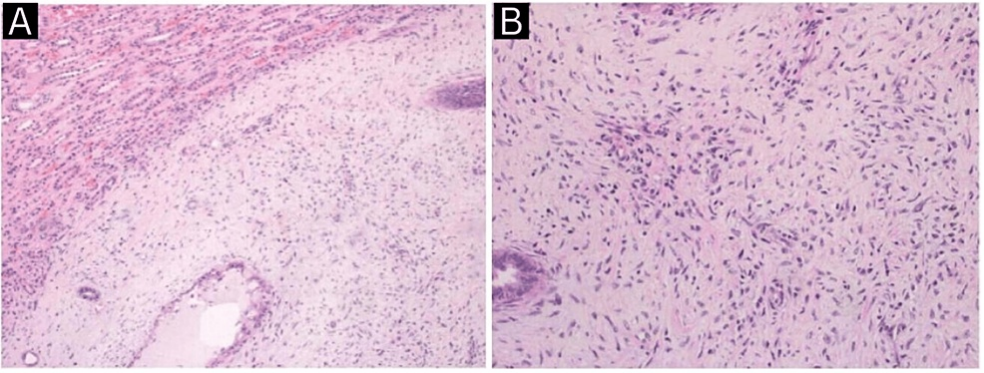
Histopathological findings (A, B) of renomedullary interstitial cell tumor.

**Figure 5:**
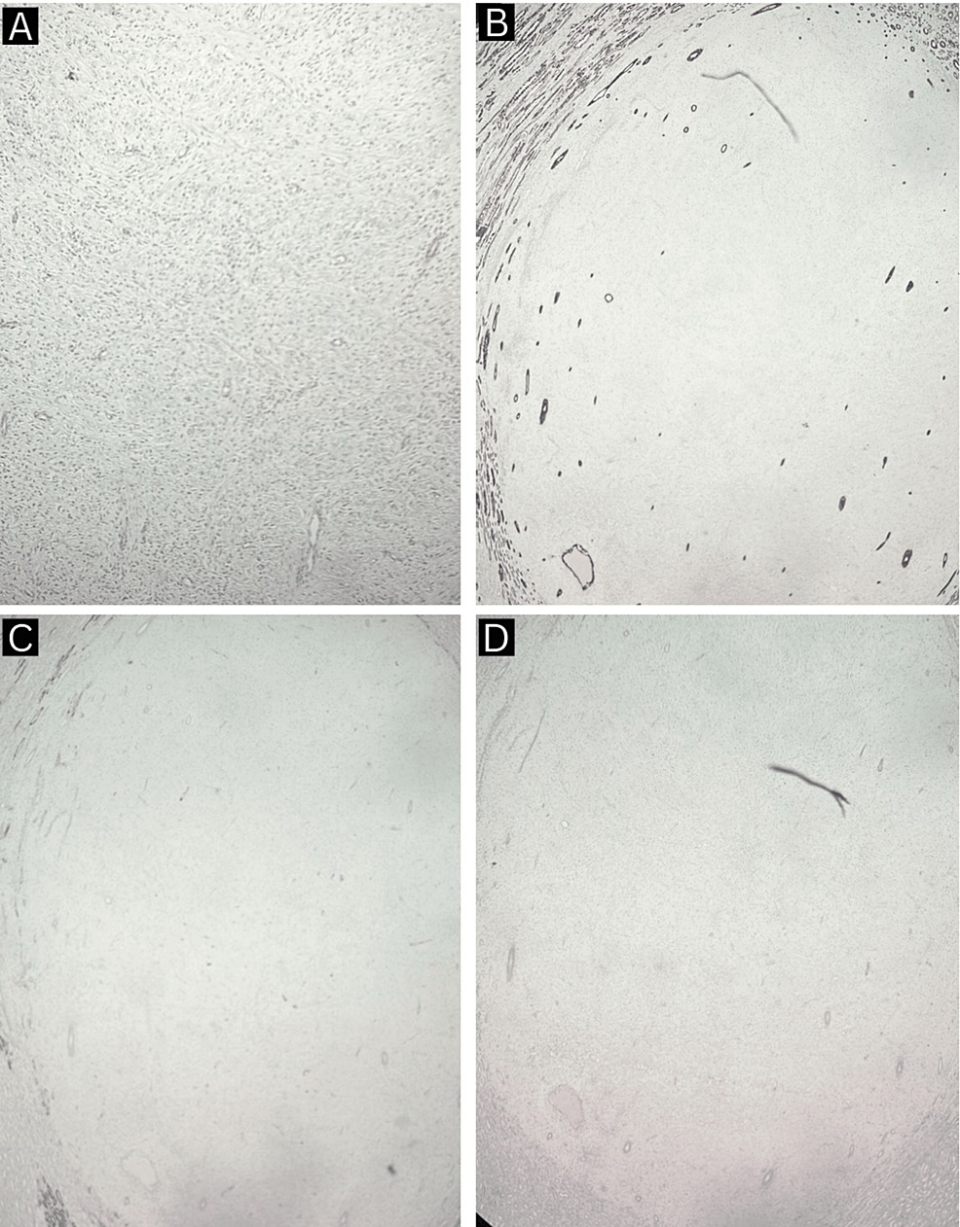
Immunohistochemistry of renomedullary interstitial cell tumor revealing CD 34 and calponin -ve cells (A), cytokeratin 7 -ve cells (B), smooth muscle actin -ve cells (C), and desmin -ve cells (D).

Another white, round smaller lesion is identified measuring 0.6 x 0.5 x 0.5 cm and is 1 cm from the main mass consistent with a RMICT (medullary fibroma) (Figures 4, 5).

The post-operative length of stay was two days and unremarkable. The patient was then discharged in good condition.

## Discussion

RMICTs are benign tumors that originate from the interstitial cells within the renal medulla, specifically located in the inner medulla. These cells exhibit receptors for various vasoactive peptides. Renomedullary interstitial cells (RMICs) assume a crucial role in the release of renin and the regulation of sodium excretion, thereby contributing significantly to the maintenance of renal blood flow and normal blood pressure. Clinically, RMICTs are frequently encountered as incidental discoveries during autopsies, with a reported prevalence ranging from 16% to 41.8% or post nephrectomies that were conducted for other tumors making their association with the overall prognosis if they manifest in the setting of another tumor (renal oncocytoma in our case) trivial [4]. Additionally, there are occasional instances of their identification through needle biopsy. However, rare instances have been observed where patients present with hematuria, hypertension, hydronephrosis, or urosepsis [5]. Patients with RMICTs have no significant identifiable risk factors such as advanced age, smoking, diabetes, or hypertension compared to those without the tumor [6]. Radiologically, RMICTs are homogeneous, commonly solitary, but can rarely manifest as multiple or bilateral. Their size can vary within the range of 1 mm to 1 cm [7]. However, giant RMICTs that reached 5 cm have been reported [8]. Due to their origin from medullary interstitial cells, the majority of RMICs are situated within the renal medulla (0-9 mm from the collecting system). This discovery could potentially facilitate their differentiation from tumors that arise from the renal cortex, such as renal cell carcinoma [9]. Gross pathology of RMICTs appears typically as round to oval, well-defined masses with a soft texture, and a coloration ranging from tan to white. Histopathologically, RMICTs appear as monomorphic spindle/stellate cells in a background of fibrotic stroma with entrapped tubules [7]. Pathologic differential diagnosis of this tumor is crucial to distinguish them from metanephric stromal tumors, which exhibit a distinctive pattern characterized by the formation of concentric "onionskin" rings or collarettes around entrapped renal tubules and blood vessels in a myxoid background.

## Conclusions

Co-presence of a renomedullary interstitial cell tumor (RMICT) in the background of a renal oncocytoma is a rare entity that can add and guide several medical specialties towards considering such a deferential diagnosis when dealing with a patient presenting with a small renal lesion in the setting of a larger one. Moreover, the lack of significant risk factors for RMICTs makes the presentation miscellaneous in terms of identifying high-risk patients. Clinical and radiological findings in this case report demonstrate that a pathognomonic presentation does not exist as it resembles renal cell carcinoma. Pre-operative diagnosis of RMICTs is difficult and a pathologic diagnosis is required. Physicians need to carefully investigate such patients using an individualized approach to avoid any form of overtreatment.
